# Cognitive training combined with nursing care versus routine daily care in improving cognitive function and behavioral symptoms in elderly patients with dementia: A retrospective study

**DOI:** 10.1097/MD.0000000000048185

**Published:** 2026-04-10

**Authors:** Mei Liu, Ya Liu, Xiaoxia Lin, Yunlan Jiang

**Affiliations:** a College of Nursing, Chengdu University of Traditional Chinese Medicine, Chengdu, Sichuan, China; b Internal Medicine, De Yang People’s Hospital, Deyang, Sichuan, China; c Internal Medicine, Hospital of Chengdu University of Traditional Chinese Medicine, Chengdu, Sichuan, China.

**Keywords:** cognitive training, dementia, emotional recovery, nursing intervention, quality of life, retrospective study

## Abstract

Dementia is a progressive neurodegenerative disorder that severely impairs cognition and daily function among older adults. Non-pharmacological interventions, particularly cognitive training integrated with individualized nursing care, have been proposed to enhance cognitive and emotional outcomes. This study aimed to evaluate the effects of cognitive training combined with nursing care compared with routine daily care in elderly patients with dementia. A retrospective observational study was conducted among 126 dementia patients treated between November 2023 and December 2024. Patients were divided into a control group (routine care) and an intervention group (cognitive training + nursing care). Each session lasted 40 minutes, 3 times weekly for 8 weeks. Cognitive, emotional, and functional outcomes were assessed using the mini-mental state examination (MMSE), Montreal cognitive assessment, Hamilton anxiety scale and depression Scales, Barthel index of activities of daily living, and general quality of life inventory-74. Propensity-score matching and receiver operating characteristic analysis were applied. After 8 weeks, the intervention group showed significantly greater improvement in MMSE (+5.4 vs +2.6) and Montreal cognitive assessment (+5.0 vs +2.4) scores than the control group (both *P* < .001). Hamilton anxiety scale and depression scores decreased more markedly in the intervention group (−6.7 and −5.5 points, *P* < .001). Barthel index of activities of daily living and general quality of life inventory-74 scores also increased significantly (both *P* < .001). Correlation analysis revealed significant negative associations between cognitive and emotional improvements (*r* = −0.67 to −0.46, *P* < .001). Receiver operating characteristic analysis showed that ΔMMSE had a strong predictive value for emotional recovery (area under the curve = 0.93, 95% confidence interval: 0.88–0.98), with 90.4% sensitivity and 87.5% specificity. Cognitive training combined with nursing intervention significantly improved cognition, emotion, and daily function in elderly patients with dementia. Cognitive improvement strongly predicted emotional recovery, underscoring the value of integrated, person-centered dementia care models in clinical practice.

## 1. Introduction

Dementia is a progressive neurodegenerative disorder characterized by cognitive decline and impaired daily functioning. In China, approximately 6% of adults aged 60 years and older are affected, accounting for nearly 15 million individuals and representing the largest dementia population worldwide. With rapid population aging, the burden of dementia is expected to increase substantially in the coming decades, posing major challenges to healthcare systems and families. These trends underscore the urgent need for effective and scalable non-pharmacological interventions.^[1–5]^

In recent years, non-pharmacological interventions have gained increasing attention as essential components of comprehensive dementia care. Among them, cognitive training combined with nursing intervention has shown promising potential to improve cognitive function, emotional stability, and overall quality of life. Cognitive training involves structured, repetitive exercises targeting domains such as memory, attention, and executive functioning, aiming to stimulate neural plasticity and delay cognitive deterioration.^[6,7]^ When integrated with personalized nursing care, such programs can further enhance adherence, reduce anxiety and depression, and improve patient engagement through empathetic communication and psychosocial support.^[8–10]^

Accumulating evidence suggests that cognitive training interventions can significantly improve cognition, mood, and coping strategies in older adults with mild cognitive impairment or dementia.^[9,10]^ This combined, person-centered model is consistent with modern dementia care principles emphasizing individualized support, family involvement, and maintenance of dignity and autonomy. However, despite encouraging international results, research on the clinical application of combined cognitive and nursing interventions in China remains scarce, especially in community and long-term care settings.

Therefore, this study aimed to evaluate the effects of cognitive training combined with nursing intervention on cognitive function, emotional state, and anxiety in elderly patients with dementia. The findings are expected to provide empirical evidence for developing culturally appropriate, sustainable, and patient-centered nursing strategies that enhance cognitive recovery, emotional well-being, and overall care quality for individuals with dementia in China.

## 2. Methods

### 2.1. Study design and ethical considerations

This study was approved by the Ethics Committee of the Hospital of Chengdu University of Traditional Chinese Medicine. This retrospective observational study was conducted in the Department of Neurology of our hospital from November 2023 to December 2024. The study adhered to the ethical principles of the Declaration of Helsinki and was approved by the institutional ethics committee. Written informed consent was obtained from legal guardians or immediate family members of all participants. Patient privacy was strictly protected, and all data were anonymized prior to analysis. The study followed the strengthening of the reporting of observational studies in epidemiology guidelines to ensure methodological rigor, transparency, and reproducibility. The primary objective was to evaluate the effects of cognitive training combined with nursing intervention on cognitive function, emotional state, and daily living ability in elderly patients with dementia. Although this was a retrospective observational study, the cognitive training combined with nursing intervention had been established as a standardized clinical rehabilitation program in our institution prior to the initiation of this research. Intervention details, including duration, frequency, and content, were routinely documented in electronic nursing records. Patients were retrospectively grouped according to the type of care they had already received during routine clinical practice.

### 2.2. Participants

A total of 126 patients diagnosed with dementia were enrolled between November 2023 and December 2024. Diagnosis was based on the diagnostic and statistical manual of mental disorders, fifth edition criteria, and confirmed by 2 senior neurologists. Eligible participants were aged 60 to 85 years, had mild to moderate dementia with mini-mental state examination (MMSE) scores ranging from 10 to 24, and were in stable physical condition that allowed participation in cognitive training sessions. Patients with severe psychiatric or other neurodegenerative disorders, recent (within 3 months) cerebrovascular or cardiovascular events, or significant sensory or speech impairments that interfered with communication were excluded. Written informed consent was obtained from each patient's legal guardian or immediate family member. According to the type of nursing intervention received, patients were divided into 2 groups: the control group (n = 63), which received routine nursing care including medication guidance, safety management, and health education, and the intervention group (n = 63), which received structured cognitive training combined with individualized nursing care in addition to routine management.

### 2.3. Cognitive training and nursing intervention protocol

The intervention lasted 8 weeks, with 3 sessions per week, each lasting approximately 40 minutes. Sessions were conducted individually or in small groups of 3 to 5 patients in a quiet rehabilitation room by nurses and psychologists certified in dementia care.

#### 2.3.1. Cognitive training content

The program covered 6 progressive cognitive domains:

1. Orientation and attention: Orientation to time, place, and person; backward counting; visual tracking;
2. Memory: Story recall, paired-word learning, and daily event recollection;
3. Language: Object naming, reading aloud, sentence completion, and semantic categorization; Calculation and reasoning: Basic arithmetic, logical sequencing, and problem-solving tasks (e.g., shopping plan preparation);
4. Visuospatial ability: Picture matching, shape reconstruction, and simple jigsaw puzzles;
5. Executive function: Task sequencing, goal setting, and decision-making in simulated daily situations. The level of difficulty was adjusted according to baseline MMSE scores and patient tolerance. Nurses provided verbal prompts and encouragement to maintain motivation and participation.

#### 2.3.2. Nursing intervention content

The nursing component focused on emotional communication, motivational guidance, and environmental orientation. Empathetic communication and reminiscence therapy were used to alleviate anxiety and depressive emotions. Environmental cues such as clocks, name tags, and family photographs were placed in the ward to strengthen spatial and temporal orientation. Family members were encouraged to participate once a week to reinforce continuity of home care. All participating nurses received standardized dementia care training to ensure intervention consistency.

### 2.4. Outcome measures

All outcomes were assessed at baseline and after the 8-week intervention by trained evaluators blinded to group assignment.

1. Cognitive function: Evaluated using the MMSE and the Montreal cognitive assessment (MoCA) for attention, memory, and executive ability.
2. Emotional and psychological status: Assessed using the Hamilton anxiety scale (HAMA) and the Hamilton depression scale (HAMD) to quantify anxiety and depressive symptoms.
3. Functional ability and quality of life: Measured by the Barthel Index of Activities of Daily Living (ADL) and the simplified general quality of life inventory-74.
4. Nursing-related indicators: Compliance (attendance and task completion) and nursing satisfaction were assessed through structured questionnaires.
5. Correlative and composite outcomes: Pearson correlation analysis was used to examine the relationship between cognitive and emotional improvements. A composite response was defined as a ≥20% improvement in both cognitive and emotional scores after intervention.

## 3. Statistical analysis

All data were analyzed using the Statistical Package for the Social Sciences version 26.0 and MedCalc version 22.0. Continuous variables were tested for normality with the Shapiro–Wilk test and presented as mean ± standard deviation ($\bar{x}\pm s$) or median (interquartile range) as appropriate. Between-group comparisons were performed using independent-samples *t* tests or Mann–Whitney *U* tests, and within-group changes were analyzed using paired t tests or Wilcoxon signed-rank tests. Categorical variables were compared using chi-square or Fisher exact tests. Pearson correlation analysis was applied to assess associations between cognitive (ΔMMSE, ΔMoCA) and emotional (ΔHAMA, ΔHAMD) improvements. Receiver operating characteristic (ROC) analysis was used to evaluate the predictive value of cognitive improvement for emotional recovery. The area under the curve, 95% confidence interval, sensitivity, specificity, and Youden index were calculated.

## 4. Results

### 4.1. Basic characteristics

A total of 126 patients were included, with 63 in each group. Mean age was 71.5 ± 6.7 years in the experimental group and 72.0 ± 7.1 years in the control group. Males accounted for 42.9% and 46.0%, and disease duration averaged 4.8 ± 2.2 and 4.6 ± 2.3 years. Educational levels, marital status, and living conditions were similar. Baseline MMSE, MoCA, HAMA, HAMD, ADL, albumin, and Pittsburgh Sleep Quality Index scores showed no notable differences between groups. The prevalence of hypertension (47.6% vs 44.4%), diabetes (22.2% vs 25.4%), and cardiovascular disease (27.0% vs 28.6%) was comparable. (Table 1)

**Table 1:** Baseline characteristics of patients.

Variables	Experimental group (n = 63)	Control group (n = 63)	*P*
Age (y)	71.5 ± 6.7	72.0 ± 7.1	0.63
Gender, n (%)			0.84
Male	27 (42.9%)	29 (46.0%)	
Female	36 (57.1%)	34 (54.0%)	
Disease duration (y)	4.8 ± 2.2	4.6 ± 2.3	0.61
Educational level, n (%)			
Primary school or below	22 (34.9%)	18 (28.6%)	0.41
Junior/senior high school	30 (47.6%)	33 (52.4%)	0.62
College or above	11 (17.5%)	12 (19.0%)	0.81
Widowed, n (%)	25 (39.7%)	26 (41.3%)	0.86
Living arrangement (with family, n %)	55 (87.3%)	54 (85.7%)	0.77
MMSE (score)	16.3 ± 3.6	15.8 ± 3.9	0.48
MoCA (score)	15.1 ± 3.5	14.8 ± 3.7	0.58
HAMA (score)	19.2 ± 4.4	18.9 ± 4.7	0.72
HAMD (score)	17.5 ± 4.0	17.2 ± 4.3	0.69
ADL (Barthel index)	59.5 ± 9.0	60.2 ± 8.8	0.61
Nutritional status (Albumin, g/L)	38.7 ± 4.8	38.1 ± 4.9	0.57
Sleep quality (PSQI)	9.5 ± 3.2	9.1 ± 3.3	0.54
Comorbidities, n (%)			
Hypertension	30 (47.6%)	28 (44.4%)	0.68
Diabetes	14 (22.2%)	16 (25.4%)	0.71
Cardiovascular disease	17 (27.0%)	18 (28.6%)	0.83

ADL = daily living ability, HAMA = Hamilton anxiety scale, HAMD = Hamilton depression scale, MMSE = mini-mental state examination scale, MoCA = Montreal cognitive assessment, PSQI = Pittsburgh sleep quality index.

### 4.2. Changes in cognitive function

After the 8-week intervention, both groups showed significant cognitive improvement, but the effect was more pronounced in the intervention group. As shown in Table 2, MMSE scores increased from 16.4 ± 3.5 to 21.8 ± 3.1 in the intervention group and from 16.0 ± 3.7 to 18.6 ± 3.4 in the control group (*P* < .001). MoCA scores also improved significantly in both groups, with greater gains in the intervention group (*P* < .001).

**Table 2:** Comparison of cognitive function scores before and after intervention between the 2 groups ($\bar{x}\pm s$).

Variable	Time point	Intervention group (n = 63)	Control group (n = 63)	*t*	*P*
MMSE (score)	Before intervention	16.4 ± 3.5	16.0 ± 3.7	0.61	0.54
	After intervention	21.8 ± 3.1	18.6 ± 3.4	5.42	<0.001
	*t*/*p* (within-group)	10.23/<0.001	4.67/<0.001		
MoCA (score)	Before intervention	15.2 ± 3.3	15.0 ± 3.5	0.31	0.76
	After intervention	20.2 ± 3.0	17.4 ± 3.2	4.71	<0.001
	t/ p (within-group)	9.85/<0.001	4.32/<0.001		

MMSE = mini-mental state examination scale, MoCA = Montreal cognitive assessment.

### 4.3. Emotional and psychological outcomes

After the intervention, both groups exhibited significant reductions in anxiety and depressive symptoms, but the improvements were more pronounced in the intervention group. As shown in Table 3, HAMA scores decreased from 19.1 ± 4.3 to 12.4 ± 3.8 in the intervention group and from 18.9 ± 4.6 to 15.7 ± 3.9 in the control group (*P* < .001). Similarly, HAMD scores declined from 17.3 ± 3.9 to 11.8 ± 3.4 and 17.0 ± 4.2 to 14.5 ± 3.6, respectively (*P* < .001). Between-group comparisons after the intervention confirmed statistically greater reductions in both HAMA and HAMD scores in the intervention group (*P* < .001).

**Table 3:** Comparison of emotional and psychological scores before and after intervention between the 2 groups ($\bar{x}\pm s$).

Variable	Time point	Intervention group (n = 63)	Control group (n = 63)	*t*	*P*
HAMA (score)	Before intervention	19.1 ± 4.3	18.9 ± 4.6	0.26	0.79
	After intervention	12.4 ± 3.8	15.7 ± 3.9	4.69	<0.001
	*t*/*p* (within-group)	8.21/<0.001	4.10/<0.001		
HAMD (score)	Before intervention	17.3 ± 3.9	17.0 ± 4.2	0.43	0.67
	After intervention	11.8 ± 3.4	14.5 ± 3.6	4.28	<0.001
	*t*/*p* (within-group)	8.52/<0.001	4.35/<0.001		

HAMA = Hamilton anxiety rating scale, HAMD = Hamilton depression rating scale.

### 4.4. Functional and quality-of-life outcomes

Both groups showed significant postintervention improvement in functional ability and quality of life, with the intervention group achieving greater gains. As shown in Table 4, ADL and general quality of life inventory-74 scores increased markedly in both groups (*P* < .001), and between-group comparisons confirmed significantly higher postintervention scores in the intervention group (*P* < .001).

**Table 4:** Comparison of functional ability and quality-of-life scores before and after intervention between the 2 groups ($\bar{x}\pm s$)

Variable	Time point	Intervention group (n = 63)	Control group (n = 63)	*t*	*P*
ADL (Barthel index)	Before intervention	59.8 ± 8.7	60.3 ± 8.9	0.34	0.73
	After intervention	75.6 ± 7.9	68.2 ± 8.1	5.23	<0.001
	*t*/*p* (within-group)	10.02/ <0.001	5.46/ <0.001		
GQOLI-74 (score)	Before intervention	58.4 ± 6.9	58.1 ± 7.2	0.22	0.83
	After intervention	72.8 ± 6.4	66.5 ± 6.7	5.69	<0.001
	*t*/*p* (within-group)	11.12/<0.001	6.03/<0.001		

ADL = daily living ability, GQOLI-74 = general quality of life inventory-74.

### 4.5. Correlation and ROC analysis

**Figure 1.**
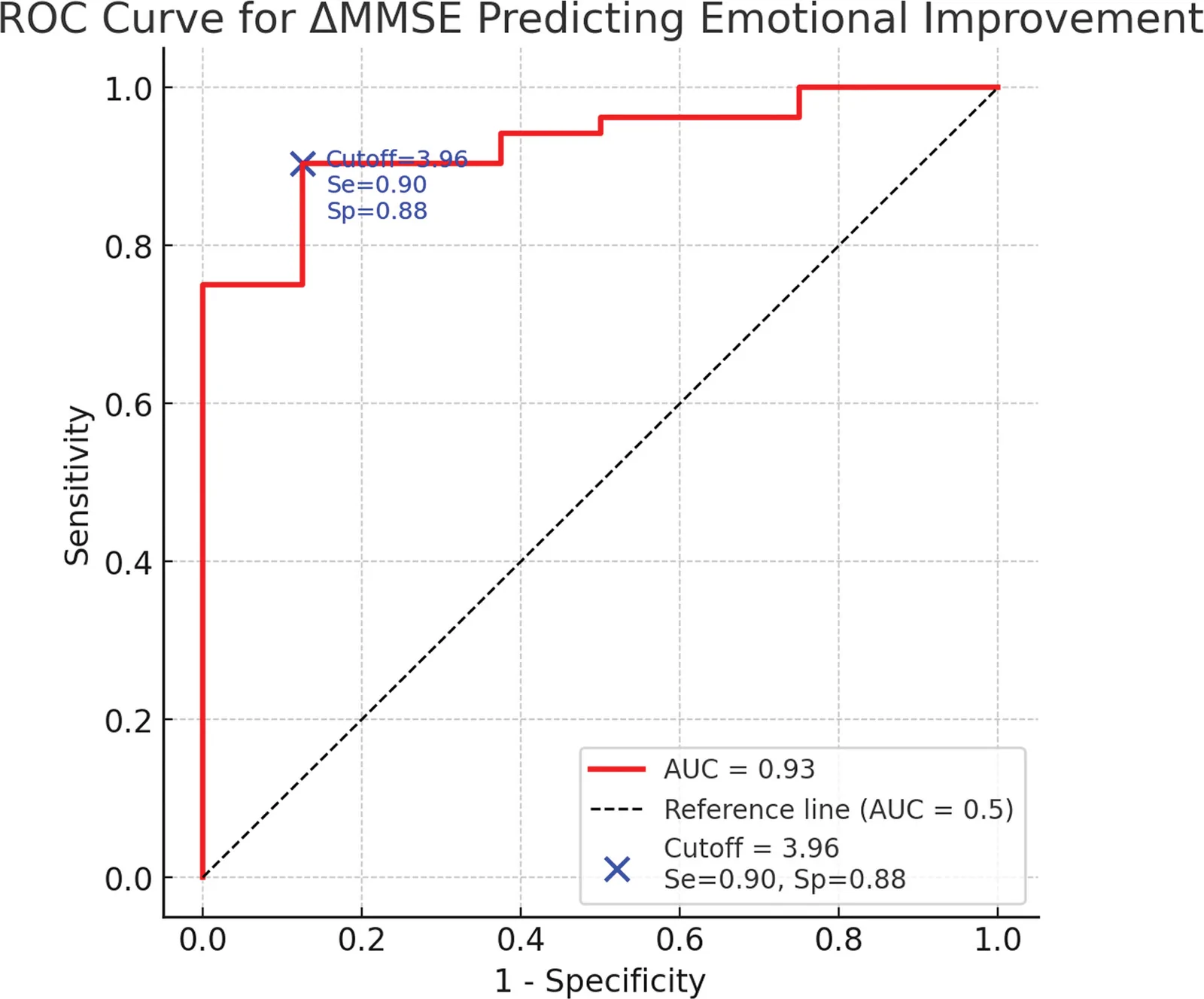
ROC curve illustrating the predictive value of cognitive improvement (ΔMMSE) for emotional recovery. AUC = area under the curve, MMSE = mini-mental state examination, ROC = receiver operating characteristic.

Correlation analysis revealed significant negative associations between cognitive and emotional improvements. Increases in MMSE and MoCA scores were moderately correlated with reductions in HAMA (*r* = −0.67 and −0.57, *P* < .001) and HAMD (*r* = −0.58 and −0.46, *P* < .001) scores (Table 5). As shown in Figure 1, ROC curve analysis demonstrated that cognitive improvement (ΔMMSE) predicted emotional recovery, with an area under the curve of 0.93 (95% confidence interval: 0.88–0.98, *P* < .001). The optimal cutoff of 3.96 points yielded 90.4% sensitivity and 87.5% specificity.

**Table 5:** Correlation between improvements in cognitive and emotional outcomes.

Variables	ΔHAMA	ΔHAMD
ΔMMSE	*r* = −0.67, *P* < .001	*r* = −0.58, *P* < .001
ΔMoCA	*r* = −0.57, *P* < .001	*r* = −0.46, *P* < .001

HAMA = Hamilton anxiety rating scale, HAMD = Hamilton depression rating scale, MMSE = mini-mental state examination, MoCA = Montreal cognitive assessment.

## 5. Discussion

This retrospective observational study demonstrated that cognitive training combined with individualized nursing intervention significantly improved cognitive function, emotional well-being, and functional ability in elderly patients. The present results are consistent with previous studies showing that structured cognitive training can enhance cognitive performance, stimulate neuroplasticity, and delay the progression of dementia.^[11–13]^ Repetitive and domain-specific exercises in attention, memory, and executive functioning reinforce residual neural networks and promote compensatory mechanisms in the aging brain.^[11,12]^ In addition, the integration of nursing interventions such as emotional communication, motivational guidance, and reminiscence therapy further enhances treatment adherence and psychological stability.^[14,15]^ These elements are known to mitigate behavioral and psychological symptoms of dementia and improve patient–caregiver interaction.^[16]^ The observed decreases in anxiety and depression levels suggest that nursing care contributes not only to emotional regulation but also indirectly to improved cognitive engagement through stress reduction and environmental familiarity.^[7]^

Several mechanisms may explain the observed benefits. Cognitive exercises improve neural activation and inter-regional connectivity, particularly within prefrontal and hippocampal circuits related to learning and memory.^[12,13]^ Supportive nursing interventions foster a sense of security and belonging, encouraging patients to participate more actively in cognitive activities.^[16]^ Family involvement also reinforces continuity of care and sustains intervention effects beyond clinical settings.^[17]^ The bidirectional relationship between cognition and emotion implies that improvement in 1 domain reinforces progress in the other, forming a positive feedback loop that enhances both psychological resilience and cognitive adaptability.^[18]^

Clinically, these findings indicate that cognitive training should be integrated into holistic dementia care programs rather than implemented as an isolated rehabilitation tool.^[11,15]^ The strong association between cognitive and emotional improvement suggests that cognitive performance may serve as a practical predictor of emotional recovery, as confirmed by ROC analysis. Monitoring cognitive changes could therefore help identify patients most likely to benefit from psychological interventions and guide personalized care planning. This multidimensional model aligns with person-centered dementia care principles, emphasizing individualized support, emotional engagement, and family participation.^[18]^ From a neuroscientific perspective, cognitive training has been shown to enhance synaptic plasticity and functional connectivity within prefrontal and hippocampal networks, which are crucial for memory, executive function, and emotional regulation. Neuroimaging studies suggest that repeated cognitive stimulation may increase neural efficiency and compensatory recruitment of preserved brain regions in patients with dementia. Moreover, emotional regulation is closely linked to prefrontal–limbic circuit function, indicating that cognitive enhancement may indirectly alleviate anxiety and depressive symptoms through shared neural pathways. The integration of supportive nursing interventions may further facilitate these neuroplastic changes by reducing stress and enhancing patient engagement.

The study also contributes new empirical evidence from a Chinese clinical context, where research on combined cognitive and nursing interventions remains limited.^[17]^ The inclusion of multidimensional outcomes: cognition, emotion, daily function, and quality of life, provides a comprehensive evaluation of intervention efficacy. Moreover, the incorporation of ROC analysis offers an innovative perspective on the predictive relationship between cognitive and emotional recovery, adding methodological rigor to non-pharmacological dementia management research.^[19]^

Several limitations of this study should be acknowledged. First, due to the retrospective observational design, selection bias cannot be entirely excluded, and unmeasured confounding factors may have influenced the observed outcomes. Second, although propensity-score matching was applied to improve baseline comparability, residual confounding may still exist. Third, the retrospective nature of the study limits causal inference between cognitive improvement and emotional recovery. Fourth, the relatively short intervention and follow-up period may not fully capture the long-term sustainability of the observed benefits. Future multicenter prospective studies with longer follow-up and randomized designs are warranted to confirm and extend these findings.

In summary, cognitive training combined with individualized nursing intervention significantly improved cognitive function, alleviated anxiety and depression, and enhanced daily functioning and quality of life among elderly patients with dementia. The strong correlation between cognitive and emotional recovery suggests that cognitive enhancement serves as a critical foundation for psychological improvement. This integrated, person-centered care model represents a practical and culturally adaptable approach to dementia management in China and holds promise for wider clinical application and future research.

## Author contributions

**Conceptualization:** Mei Liu, Ya Liu, Xiaoxia Lin, Yunlan Jiang.

**Data curation:** Mei Liu, Ya Liu, Xiaoxia Lin, Yunlan Jiang.

**Formal analysis:** Mei Liu, Ya Liu, Xiaoxia Lin, Yunlan Jiang.

**Funding acquisition:** Ya Liu, Xiaoxia Lin, Yunlan Jiang.

**Investigation:** Ya Liu, Xiaoxia Lin, Yunlan Jiang.

**Writing – original draft:** Mei Liu, Xiaoxia Lin, Yunlan Jiang.

**Writing – review & editing:** Mei Liu, Xiaoxia Lin, Yunlan Jiang.
